# Metal–Organic Frameworks Functionalized Separators for Robust Aqueous Zinc-Ion Batteries

**DOI:** 10.1007/s40820-022-00960-z

**Published:** 2022-11-09

**Authors:** Yang Song, Pengchao Ruan, Caiwang Mao, Yuxin Chang, Ling Wang, Lei Dai, Peng Zhou, Bingan Lu, Jiang Zhou, Zhangxing He

**Affiliations:** 1 School of Chemical Engineering, North China University of Science and Technology, Tangshan, 063009 People’s Republic of China; 2 School of Materials Science and Engineering, Hunan Provincial Key Laboratory of Electronic Packaging and Advanced Functional Materials, Central South University, Changsha, 410083 People’s Republic of China; 3 Hunan Provincial Key Defense Laboratory of High Temperature Wear-Resisting Materials and Preparation Technology, Hunan University of Science and Technology, Xiangtan, 411201 People’s Republic of China; 4 School of Physics and Electronics, Hunan University, Changsha, 410082 People’s Republic of China

**Keywords:** Aqueous zinc-ion batteries, Separators, Metal–organic frameworks, Ion transport, Dendrite-free

## Abstract

Aqueous zinc-ion batteries (AZIBs) are one of the promising energy storage systems, which consist of electrode materials, electrolyte, and separator. The first two have been significantly received ample development, while the prominent role of the separators in manipulating the stability of the electrode has not attracted sufficient attention. In this work, a separator (UiO-66-GF) modified by Zr-based metal organic framework for robust AZIBs is proposed. UiO-66-GF effectively enhances the transport ability of charge carriers and demonstrates preferential orientation of (002) crystal plane, which is favorable for corrosion resistance and dendrite-free zinc deposition. Consequently, Zn|UiO-66-GF-2.2|Zn cells exhibit highly reversible plating/stripping behavior with long cycle life over 1650 h at 2.0 mA cm^−2^, and Zn|UiO-66-GF-2.2|MnO_2_ cells show excellent long-term stability with capacity retention of 85% after 1000 cycles. The reasonable design and application of multifunctional metal organic frameworks modified separators provide useful guidance for constructing durable AZIBs.

**Supplementary Information:**

The online version contains supplementary material available at 10.1007/s40820-022-00960-z.

## Highlights

- Metal-organic frameworks (UiO-66) functionalized glass fiber separator was constructed to accelerate the transport of charge carriers and provide a uniform electric field distribution on the surface of zinc anode.
- Zinc anode demonstrates preferential orientation of (002) plane under the control of UiO-66-GF, which effectively inhibits dendrites.
- Density functional theory calculation confirms that the adsorption effect of (002) plane on H is weaker, thus improving corrosion resistance and suppressing the hydrogen evolution reaction.
- Symmetric cells exhibit highly reversible plating/stripping behavior with long cycle life over 1650 h and full cells demonstrate excellent long-term stability (85%) for 1000 cycles.

**Supplementary Information:**

The online version contains supplementary material available at 10.1007/s40820-022-00960-z.

## Graphical abstract

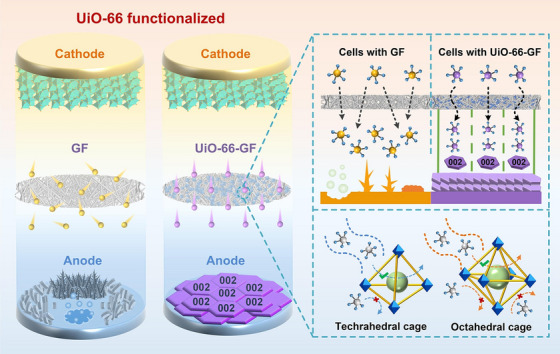

## Introduction

Aqueous zinc-ion batteries (AZIBs) have a high application potential, owing to their simple fabrication process, intrinsic safety, and economic feasibility, for a new generation of energy storage devices [1–3]. However, numerous challenges impede their practical application, particularly the inevitable issues in zinc anode, including dendrites, hydrogen evolution reaction (HER), corrosion, and passivation [4–6]. The formation and growth of dendrites generated by inhomogeneous zinc plating destroy anode–electrolyte interface and even induce short circuit, resulting in a short cycle life and poor electrochemical performance [7, 8]. Most of the current modification studies focus on the interfacial modification or structural design of zinc anode and optimal configuration of electrolyte additives to regulate the plating/stripping behavior of zinc-ions [9]. As a key part of AZIBs, separator plays a crucial role in ions transport and electrolyte carriage. The research on separators is still in its infancy, indicating that its application potential and research value need to be developed urgently [10, 11].

Separator acts to transport ions and prevent physical contact between cathode and anode. However, voids with different sizes in glass fiber (GF) are the dominant separator in AZIBs, triggering an inhomogeneous deposition of zinc-ions and dendrite growth, eventually causing a short circuit. Inspired by lithium-ion batteries (LIBs), various multi-functional materials including graphene oxide (GO) layer [12], polypyrrole (PPy) layer [13], and Sn coating [14] have been used in the separators for uniform zinc deposition. The large specific surface area of the intermediate layer enhances the reaction kinetics, and the good zinc affinity makes the zinc-ions flux uniform. Janus separator obtained by vertically growing graphene on GF has large surface area and three-dimensional (3D) framework, which is favorable for the uniform deposition of zinc-ions, thereby suppressing the formation of dendrites [15]. To compensate for the defect of nonuniform void size of GF, functional supramolecules [16] and BaTiO_3_ [17] were introduced into GF by vacuum filtration. This not only effectively accelerates the transmission of zinc-ions, but also uniformly distributes zinc-ions to the separator-zinc anode interface for highly reversible plating/stripping. To reduce the working cost and simplify the preparation process, new cost-effective separators, such as weighing paper (WP) [18] and commercial cotton towel (CT) [19], adsorb zinc-ions through their plenteous functional groups to enhance the reversibility of zinc anode. Metal–organic frameworks (MOFs) with large specific surface areas and topological structures are ideal materials for fabricating high-performance separators and have been applied in studies on lithium-sulfur (Li–S) batteries [20]. However, their excellent ion transport ability has not been embodied in AZIBs.

**Fig. 1:**
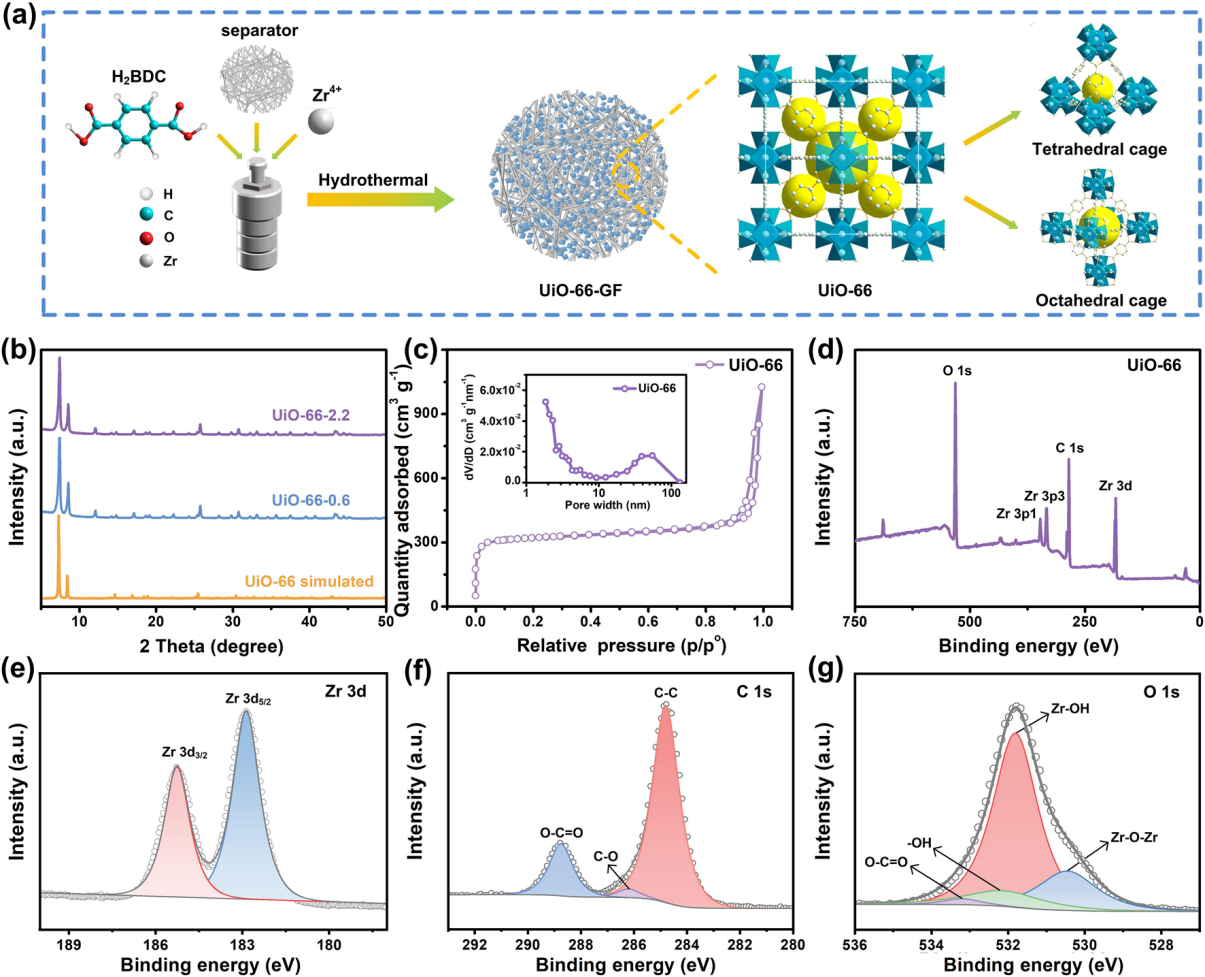
Synthesis of UiO-66-GF and characterizations of UiO-66. **a** Preparation diagram of UiO-66-GF and structural diagram of UiO-66. **b** XRD patterns of UiO-66. **c** N_2_ adsorption/desorption isotherm and pore size distribution of UiO-66. **d** XPS full spectrum of UiO-66. High-resolution XPS spectra of **e** Zr 3*d*, **f** C 1*s*, and **g** O 1*s*

In this work, we prepared a separator functionalized by a Zr-based MOF (UiO-66-GF) via a hydrothermal method, used in high-performance AZIBs (Fig. 1a). UiO-66 exhibits structural robustness. The strong Zr-O bond coordination contributes to its stability under thermal, chemical, and aqueous conditions, which is the major advantage over other MOFs materials [21]. The rich Lewis acidic sites and channels in UiO-66 also enhance the ion transport ability [22]. The large specific surface area and abundant pore structure of UiO-66 provide UiO-66-GF with high transport ability for charge carriers at separator–electrolyte interface. UiO-66 induces preferential orientation of (002) crystal plane [23], which is conducive to the growth of zinc-ions in the horizontal direction without dendrites [24]. Furthermore, undesirable side reactions, including corrosion and HER, are significantly suppressed, mainly manifested by the reduction of by-products on the zinc anode surface. Zn|UiO-66-GF-2.2|Zn cell enables over 1650 h of reversible plating/stripping with high Coulombic efficiency (CE) and low polarization (39 mV) [25]. In addition, Zn|UiO-66-GF-2.2|MnO_2_ cell exhibits high specific discharge capacity of 230.8 mAh g^−1^ at 0.1 A g^−1^ and excellent long-term stability with capacity retention of 85% after 1000 cycles at 1.0 A g^−1^. This work provides a new concept for the construction of stable zinc anode and durable AZIBs [26].

## Experimental

### Materials

Glass fiber separators were purchased from Tianjin Aiweixin Chemical Technology Co., Ltd. Terephthalic acid (H_2_BDC) was purchased from J&K Scientific Ltd. ZrCl_4_ was purchased from Shanghai Aladdin Biochemical Technology Co., Ltd. Other chemical substances were of analytical grade and had not undergone other treatments.

### Preparation of Materials

All glass fiber separators were ultrasonically treated with absolute ethanol for 0.5 h to clean the impurities on the surface and ensure the accuracy of the experimental data. UiO-66 was synthesized by hydrothermal method. Firstly, 0.6 and 2.2 mmol L^−1^ of ZrCl_4_ (0.14 and 0.513 g) were added to a beaker containing 40 mL of N, N dimethylformamide (DMF), respectively. H_2_BDC (0.1 and 0.365 g) and 4 mL of acetic acid were then added to the mixed solution, respectively. Finally, ultrasonic treatment was performed for 0.5 h. Glass fiber separators were added to the above solution, soaked for 10 min, transferred to a 100 mL of Teflon-lined stainless-steel autoclave, and heated in an oven set at 120 °C for 16 h. When the hydrothermal reaction was completed and the temperature was cooled to 25 °C, glass fiber separators were washed with methanol and placed in a vacuum drying oven at 80 °C for 8 h. The white solution in the stainless-steel autoclave was centrifuged with methanol and dried at 80 °C for 8 h to obtain a white powder UiO-66. According to the amount of ZrCl_4_ (0.6 and 2.2 mmol L^−1^), the obtained MOFs are denoted as UiO-66-0.6 and UiO-66-2.2, respectively. The original glass fiber separator is denoted as GF. The obtained MOFs in situ grown glass fiber separators are denoted as UiO-66-GF-0.6 and UiO-66-GF-2.2, respectively.

0.3803 g MnSO_4_·H_2_O and 0.237 g KMnO_4_ were added to 15 mL of distilled water and stirred for 15 min until they were completely dissolved. The above KMnO_4_ solution was then added dropwise to MnSO_4_·H_2_O. After stirring for 30 min, the mixed solution was transferred to a 100 mL Teflon-lined stainless-steel autoclave and heated at 160 °C for 12 h. After natural cooling, the resulting precipitate was centrifuged three times with distilled water and then placed in a vacuum drying oven at 80 °C to dry for 8 h. The obtained α-MnO_2_ powder was used as cathode material. α-MnO_2_, Super P, and polyvinylidene fluoride (PVDF) were mixed in a ratio of 7:2:1 with N-methyl pyrrolidone (NMP) as the solvent. After the slurry was formed, it was coated on a metal mesh (*Φ* = 14 mm) and placed in a vacuum drying oven at 80 °C for 8 h.

### Characterizations

The crystal structures of the samples were studied by X-ray diffraction (XRD, D8 Advance A25 Instrument, Bruker, Germany). Morphology was observed by scanning electron microscopy (SEM, JSM-IT100, JEOL, Japan), and energy-dispersive X-ray (EDX) analysis was carried out to analyze the surface elemental composition. X-ray photoelectron spectroscopy (XPS, K-alpha Plus Instrument, Thermo Fisher, USA) was carried out to study surface chemical states. Distilled water was used as the test liquid to test the hydrophilicity of the sample by contact angle tester (HARKE-SPCA, Beijing Hake Test Instrument Factory, China). The surface areas of the samples, degassed at 120 °C for 24 h under vacuum, were evaluated using N_2_ adsorption/desorption isotherms at − 196 °C (BET, 3H-2000PM1, BSD Instrument, China). Molecular structures and functional group types were analyzed by Fourier transform infrared spectroscopy (FTIR, VERTEX 80v, Bruker, Germany).

### Electrochemical Measurements

All CR2016 coin cells were assembled in air. Full cell was assembled with zinc foil as anode, α-MnO_2_ as cathode, and aqueous solution of 2.0 mol L^−1^ ZnSO_4_ + 0.1 mol L^−1^ MnSO_4_ as electrolyte. Zinc foil was used as anode and cathode, and 2.0 mol L^−1^ ZnSO_4_ aqueous solution was used as an electrolyte to assemble symmetrical cell. Asymmetric cells were assembled with copper foil and titanium foil as cathode, zinc foil as anode, and 2.0 mol L^−1^ ZnSO_4_ aqueous solution as electrolyte. All cells were placed on LAND test system (CT2001A, Wuhan Lanhe, China) for 4 h before constant current charge–discharge. Rate performances of full cells were analyzed at current densities of 0.1, 0.3, 0.5, 1.0, 1.2, 1.5, 2.0, 4.0, and 0.1 A g^−1^. Cycling performances were analyzed at current densities of 0.5 and 1.0 A g^−1^. Galvanostatic intermittent titration technique (GITT) was performed on LAND test system. Cells were cycled 10 times at 0.5 A g^−1^ to maintain stability. The current pulse was lasted for 10 min at 0.1 A g^−1^, and then cells were relaxed for 30 min to bring the voltage to equilibrium. Rate performances of symmetric cells were analyzed at current densities of 0.25, 0.5, 1.0, 2.0, and 4.0 mA cm^−2^. Nucleation overpotential (NOP) and Coulombic efficiency (CE) were measured by asymmetric cells at 2.0 mA cm^−2^. Chronoamperogram (CA), linear polarization test, cyclic voltammetry (CV), and electrochemical impedance spectroscopy (EIS) were measured by electrochemical workstation (CHI660E, Shanghai Chenhua, China). CA test was performed at a scan rate of 5 mV s^−1^ in 2.0 mol L^−1^ ZnSO_4_ solution, and linear polarization test was performed at a scan rate of 10 mV s^−1^. The ionic conductivities (σ) of stainless steel (SS)|GF|SS, SS|UiO-66-GF-0.6|SS, and SS|UiO-66-GF-2.2|SS cells were tested by EIS in the frequency range from 0.1 to 100,000 Hz using an electrochemical workstation (CHI660E, Shanghai Chenhua, China). The ionic conductivity was calculated by σ = d/*R*_b_S*S*, where d is the thickness of the separator and *R*_b_ and *S* represent the bulk resistance and the effective area of the separator, respectively. CV test of full cell was carried out in a range of 0.8–1.8 V at a scan rate of 0.1 mV s^−1^. CV test of Zn//Ti asymmetric cell was carried out at a scan rate of 0.5 mV s^−1^. EIS test was carried out in a range of 0.01–100,000 Hz.

### Density Functional Theory (DFT) Calculation

DFT simulations were performed using the software Visualization for Electronic and Structural Analysis (VESTA). In our calculations, we use a 7 × 7 × 7 k-point mesh for Zn optimization, while constructing a *p* (3 × 3 × 2) supercell of Zn. The adsorption energy (*E*_abs_) of Zn atom on Zn (002), (100), and (101) planes was calculated by *E*_abs_ = *E*_Zn-H_ − *E*_H_ − *E*_Zn_, where *E*_Zn-H_, *E*_H_, and *E*_Zn_ are the energy after Zn adsorbs H, energy of a single H, and energy without H adsorption, respectively. Hydrogen adsorption Δ*G*_H_ was calculated by Δ*G*_H_ = Δ*E*_DFT_ + Δ*E*_ZPE_ − *T*Δ*S*, where Δ*E*_DFT_, Δ*E*_ZPE_, and *T*Δ*S* denote the DFT calculated adsorption energy, change of zero point energy, and change of entropic contribution, respectively. *TS* term for H adsorbate is considered negligible, and *T*Δ*S* ≈ − 0.5 *S*$S_{{\text{H}}_{2}}$ = − 0.24 eV.

## Results and Discussion

### Synthesis of UiO-66-GF and Characterizations of UiO-66

As illustrated in Figs. S1 and S2, UiO-66 with a face-centered cubic crystal structure has a diameter of approximately 70 nm. The distributions of C, O, and Zr elements are consistent with the positions of SEM image. Each zirconium metal center is linked to 12 benzene-1,4-dicarboxylates (BDC) to form a 3D framework, which is favorable for its stable existence in GF [27]. According to the amount of ZrCl_4_ (0.6 and 2.2 mmol L^−1^) used in the synthesis process, the obtained MOFs are denoted as UiO-66-0.6 and UiO-66-2.2, respectively. Furthermore, UiO-66-0.6 and UiO-66-2.2 are in good agreement with XRD pattern (UiO-66 simulated) obtained by UiO-66 crystal structure parameter simulation (Fig. 1b). Characteristic diffraction peaks of UiO-66 at 7.3°, 8.5°, and 25.6° are consistent with the reported results, which demonstrates the successful synthesis of UiO-66 [28]. There is a sharp peak with weak intensity at 12.0°, which is attributed to the residual solvent [29]. Figure 1c presents a reversible type I isotherm without hysteresis, which corresponds to the typical microporous structure of MOFs. The large specific surface area (990.3 m^2^ g^−1^) and porous structure of UiO-66 provide more transport channels to facilitate the migration and diffusion of zinc-ions. As shown in Fig. 1d, the signals of C 1*s*, O 1*s*, Zr 3*d*, and Zr 3*p* are detected in the XPS full spectrum, further implying the successful synthesis of UiO-66 [30]. The high-resolution XPS spectrum of Zr 3*d* of UiO-66 in Fig. 1e exhibits corresponding peaks of Zr 3*d*_5/2_ and Zr 3*d*_3/2_ at 182.6 and 185.1 eV, respectively, which indicates that the Zr element in UiO-66 exists in the form of ZrO_2_ [31]. The C 1*s* spectrum has three peaks including those of C–C (284.8 eV), C–O (285.9 eV), and O–C=O (288.8 eV) (Fig. 1f) [32], and O 1*s* spectrum has four distinct peaks at 530.4, 531.9, 532.2, and 533.2 eV, corresponding to Zr–O–Zr, Zr–OH, –OH, and O–C=O, respectively (Fig. 1g) [33].

### Characterizations of UiO-66-GF

**Fig. 2:**
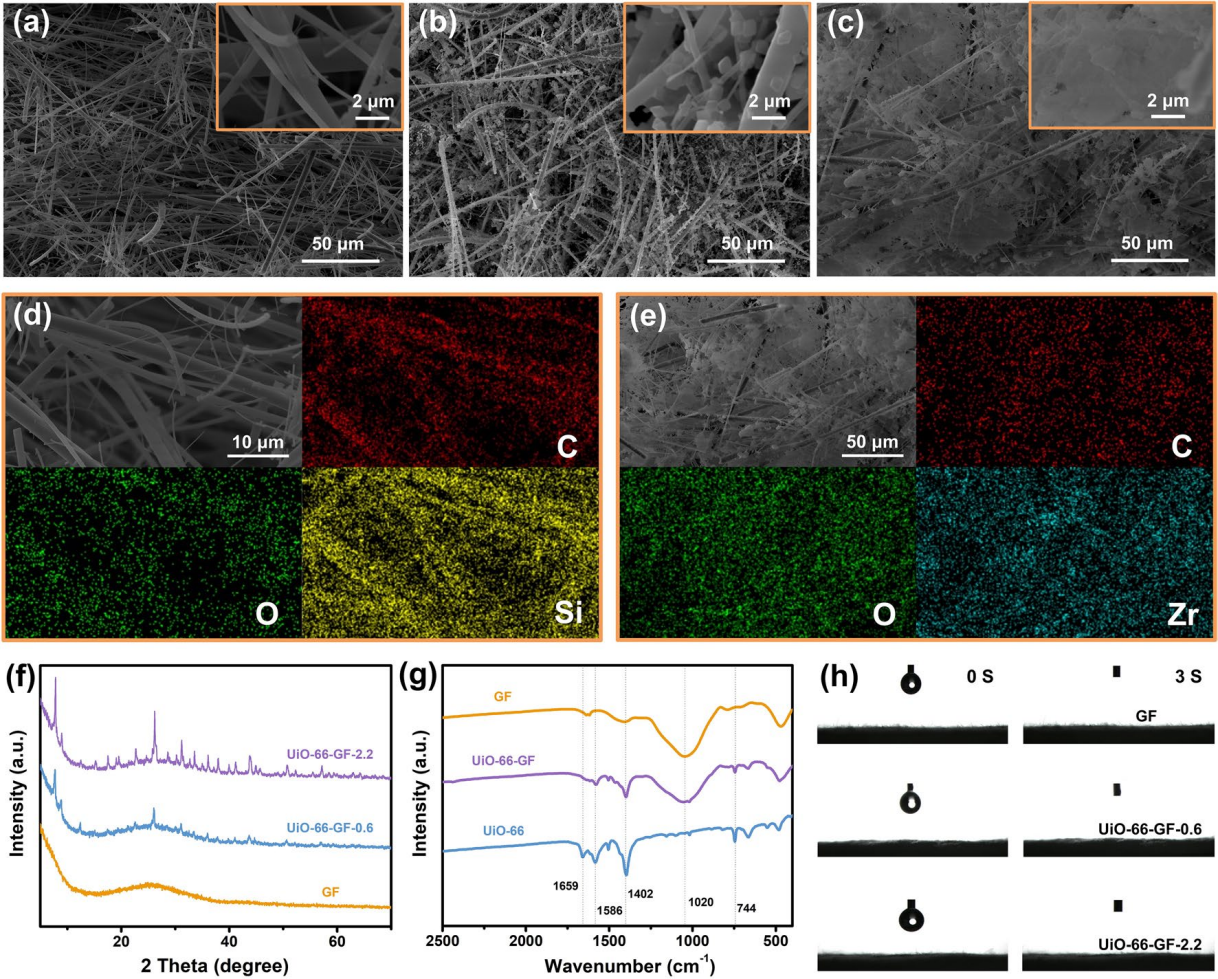
Characterizations of UiO-66-GF. SEM images of **a** GF, **b** UiO-66-GF-0.6, and **c** UiO-66-GF-2.2 at different magnifications. **d** SEM image of GF and element mapping for C, O, and Si. **e** SEM image of UiO-66-GF-2.2 and element mapping for C, O, and Zr. **f** XRD patterns of GF, UiO-66-GF-0.6, and UiO-66-GF-2.2. **g** FTIR spectra of GF, UiO-66-GF, and UiO-66. **h** Contact angle tests for three separators after 0 and 3 s

Due to the poor affinity and attraction for zinc-ions, GF is incapable of inhibiting the concentrated and disordered Zn deposition on the electrodes [16]. Moreover, although abundant porous space on the surface of GF provides a prerequisite for a rapid penetration of electrolyte (Fig. 2a), uneven distribution of porous space still limits the uniform transport of carriers, which is not conducive to the uniform plating/stripping of zinc anode, thus facilitating the formation of dendrites. Sparsely grown MOFs in UiO-66-GF-0.6 provide inadequate ion transport channels, limiting the effect of inducing uniform deposition of zinc-ions (Fig. 2b). On the contrary, MOFs inside UiO-66-GF-2.2 are uniform and can fill the voids with different sizes in GF (Fig. 2c), making the flux of zinc-ions uniform. Therefore, the uniform Zn plating layers are obtained instead of dendrites. All elements of GF are consistent with SEM image position (Figs. 2d and S3a-d). C, O, and Zr elements can also be observed in UiO-66-GF-0.6 and UiO-66-GF-2.2 (Fig. 2e and S3e–j). Moreover, significant UiO-66 diffraction peaks are observed for UiO-66-GF-0.6 and UiO-66-GF-2.2 (Fig. 2f). The peak intensity increases with concentration of the solution, which demonstrates the successful synthesis of UiO-66-GF. In the FTIR spectra of GF (Fig. 2g), the peak at 1020 cm^−1^ is ascribed to the asymmetric stretching vibration of Si–O–Si [34]. Among the diffraction peaks of UiO-66, the peak at 744 cm^−1^ corresponds to the characteristic peak of Zr–O–Zr, and the peaks at 1402, 1586, and 1659 cm^−1^ correspond to the vibrational peaks of aromatic benzene ring, respectively [35]. In addition, these peaks are also detected in UiO-66-GF, reflecting the perfect combination of UiO-66 and GF. When the electrolyte droplets reach different surfaces, droplets can be fully absorbed in 3 s, indicating that the surfaces of UiO-66-GF still maintain good wettability (Fig. 2h).

### Enhancements in Stability and Reversibility by UiO-66-GF

**Fig. 3:**
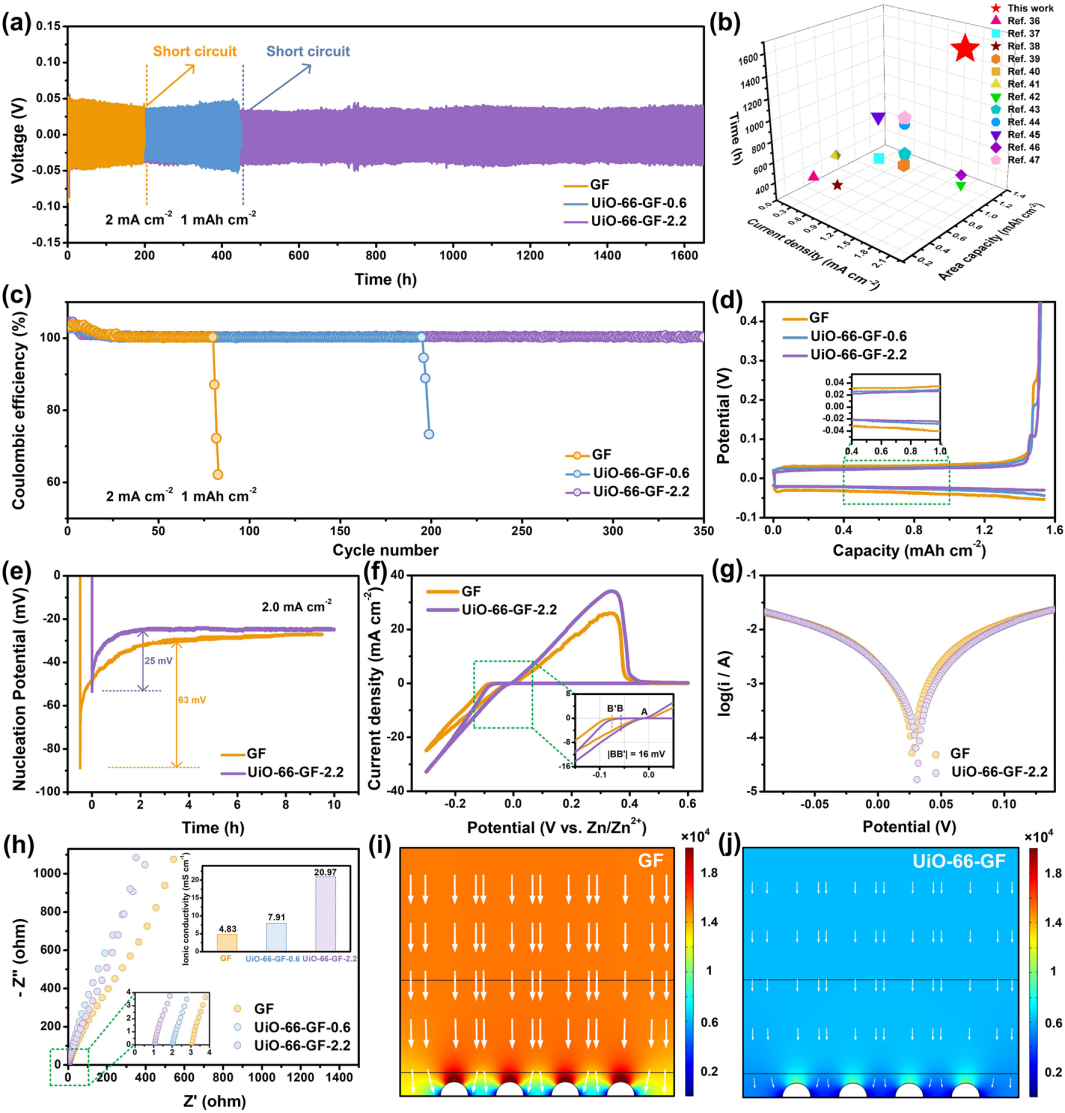
Enhancements in stability and reversibility by UiO-66-GF. **a** Galvanostatic charge/discharge cycling voltage profiles of Zn|GF|Zn, Zn|UiO-66-GF-0.6|Zn, and Zn|UiO-66-GF-2.2|Zn cells at a current density of 2.0 mA cm^−2^ for 1.0 mAh cm^−2^. **b** Comparison of cyclic reversibility obtained in this work and previous studies. **c** CE plots of three cells at a current density of 2.0 mA cm^−2^ with a capacity of 1.0 mAh cm^−2^. **d** Corresponding plating/stripping profiles of three cells at the 50^th^ cycle. **e** NOPs of Zn|GF|Cu and Zn|UiO-66-GF-2.2|Cu cells. **f** CV curves of Zn|GF|Ti and Zn|UiO-66-GF-2.2|Ti cells at 0.5 mV s^−1^. **g** Linear polarization curves of Zn|GF|Zn and Zn|UiO-66-GF-2.2|Zn cells. **h** EIS of SS|GF|SS, SS|UiO-66-GF-0.6|SS, and SS|UiO-66-GF-2.2|SS cells for the calculation of ionic conductivities. The electrical field models based on **i** GF and **j** UiO-66-GF

To verify the effectiveness of UiO-66-GF, long-term plating/stripping performances of Zn|GF|Zn, Zn|UiO-66-GF-0.6|Zn, and Zn|UiO-66-GF-2.2|Zn cells were compared. At 0.5 mA cm^−2^, Zn|GF|Zn cell suffers from serious polarization at initial phase with poor cycling stability of 200 h (Fig. S4). Zn|UiO-66-GF-0.6|Zn cell runs for 420 h, while Zn|UiO-66-GF-2.2|Zn cell can work stably for 1000 h without considerable voltage fluctuation, along with the smaller overpotential compared with Zn|GF|Zn cell (33 *vs*. 56 mV). When the current density increases to 2.0 mA cm^−2^, Zn|UiO-66-GF-2.2|Zn cell still maintains the cycling stability for more than 1650 h (Fig. 3a), with a lower overpotential of 39 mV, while Zn|GF|Zn cell is short-circuited after 195 h. Although other studies in this area demonstrate good performances, the design in this work is more efficient and profound (Fig. 3b) [36–47]. Meanwhile, hysteresis voltage of Zn|UiO-66-GF-2.2|Zn cell is always lower than that of Zn|GF|Zn cell (Fig. S5), favorable for uniform nucleation of zinc-ions [48]. Rate performances of symmetric cells at various current densities were compared to evaluate the effect of UiO-66-GF on reaction kinetics of zinc plating/stripping. As revealed by Fig. S6, polarization curves keep steady in each 20 cycles test. As current density increases from 0.25 to 4.0 mA cm^−2^, corresponding polarization voltage displays a minor increase from 56 to 82 mV for Zn|UiO-66-GF-2.2|Zn cell, which is considerably lower than those of Zn|GF|Zn and Zn|UiO-66-GF-0.6|Zn cells, indicating a stable and reversible zinc anode provided by UiO-66-GF-2.2. CEs of asymmetric cells were tested to investigate the persistence and reversibility for zinc plating/stripping. As expected, Zn|UiO-66-GF-2.2|Cu cell shows longer cycle life (350 cycles) along with lower polarization and better reversibility at 2.0 mA cm^−2^, compared with Zn|GF|Cu cell (80 cycles) and Zn|UiO-66-GF-0.6|Cu cell (190 cycles) (Fig. 3c, d) [49]. A lower NOP corresponds to a more stable and uniform zinc plating/stripping process and longer cycle life of cell [50]. The NOP of Zn|UiO-66-GF-2.2|Cu cell is 25 mV at 2.0 mA cm^−2^, lower than that of Zn|GF|Cu cell (63 mV), demonstrating that UiO-66-GF can reduce the deposition barrier of zinc-ions (Fig. 3e) [51]. Cyclic voltammetry (CV) curves of Zn|GF|Ti and Zn|UiO-66-GF-2.2|Ti cells exhibit similar oxidation and reduction peaks, and the potential difference between A and B (B') is NOP (Fig. 3f). Compared with Zn|GF|Ti cell, NOP of Zn|UiO-66-GF-2.2|Ti cell is reduced by 16 mV, displaying that UiO-66-GF-2.2 effectively reduces the deposition barrier of zinc-ions [52], which is consistent with the results of Fig. 3e.

The corrosion protections of GF and UiO-66-GF for zinc anode were analyzed by linear polarization test, directly reflected by the corrosion current (Figs. 3g and S7). The corrosion currents of Zn|GF|Zn, Zn|UiO-66-GF-0.6|Zn, and Zn|UiO-66-GF-2.2|Zn cells are 1.4, 1.0, and 0.9 mA cm^−2^, respectively. These results can be explained as UiO-66-GF regulates the flux of zinc-ions and prevents a massive aggregation of cations on zinc anode by inhibiting concentration polarization and reduces the space charge and surface barrier to accelerate the transport kinetics of zinc-ions on electrode surface [53]. Furthermore, UiO-66-GF can effectively promote charge carrier transport, as confirmed by EIS. The ionic conductivities of SS|GF|SS, SS|UiO-66-GF-0.6|SS, and SS|UiO-66-GF-2.2|SS cells are 4.83, 7.91, and 20.97 mS cm^−1^, respectively, which can be attributed to the ultra-large specific surface area of UiO-66 yielding an excellent transport process (Fig. 3h) [54]. COMSOL finite-element simulations were performed to illustrate the role of UiO-66-GF in regulating the interfacial electric field. Zinc anode surface with GF exhibits a non-uniformly distributed electric field and the increasing field strength leads to the continuous accumulation of charges (Fig. 3i), promoting the preferential deposition of more zinc-ions at the tip and the final formation of dendrites. When UiO-66-GF was employed, electric field of zinc anode surface was uniform (Fig. 3j), helping to achieve a uniform plating/stripping process [55]. This result is consistent with the structure of zinc anode for Zn|UiO-66-GF-2.2|Zn cell has a neat and smooth surface and cross section after cycling (Fig. S8). The mechanism of zinc deposition behavior can be verified by chronoamperometry (CA) tests (Fig. S9), where the two-dimension (2D) diffusion process of zinc-ions in Zn|GF|Zn cell is long and intense, corresponding to inhomogeneous zinc nucleation [56]. In contrast, Zn|UiO-66-GF-0.6|Zn and Zn|UiO-66-GF-2.2|Zn cells enter a stable 3D diffusion process after 30 s of planar diffusion and nucleation, which indicates that zinc ions are diffused uniformly and grow, likely as the confinement effect of UiO-66 inhibits the formation of dendrites [57].

### Electrochemical Performances of Full Cells

**Fig. 4:**
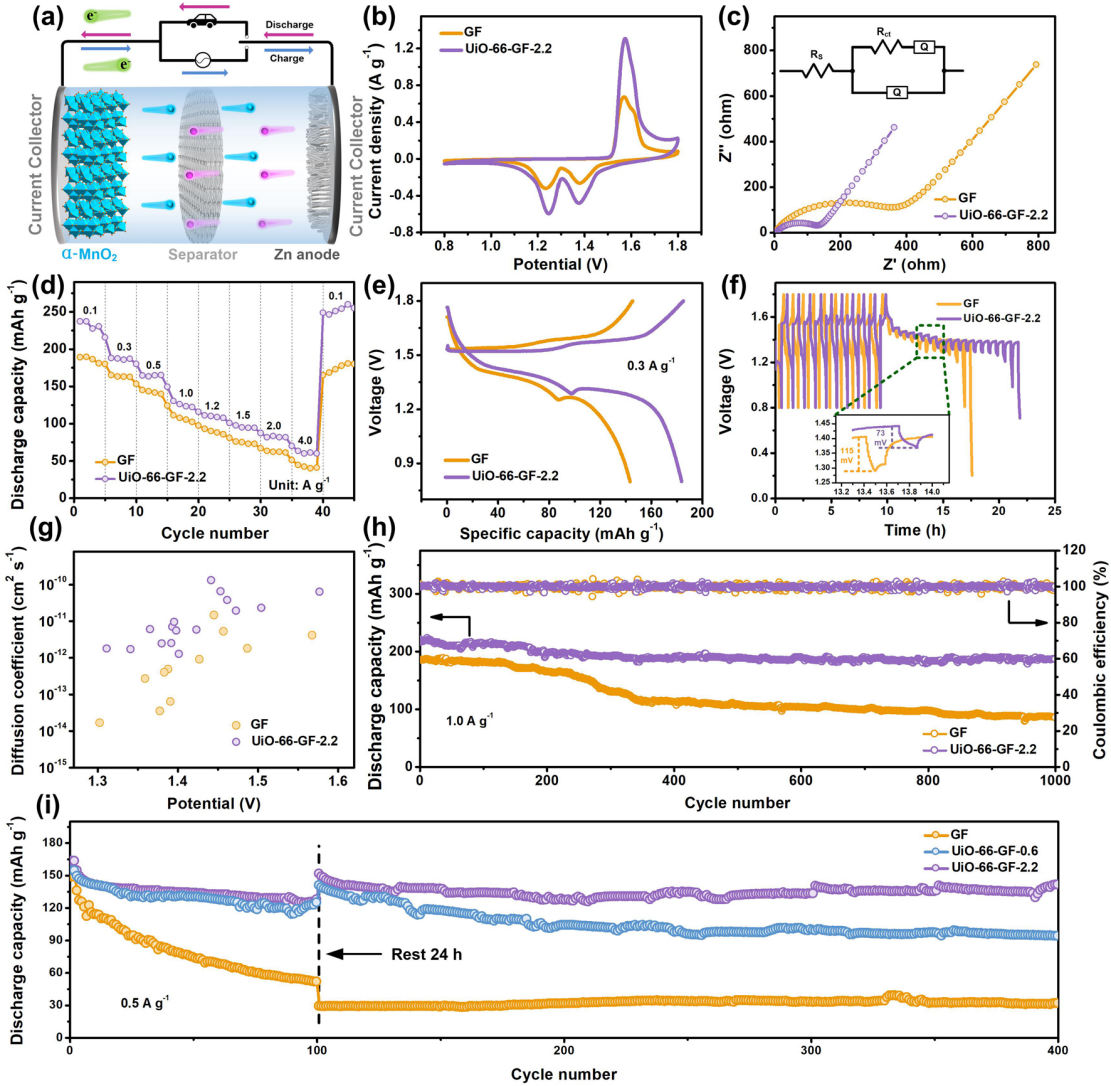
Electrochemical performances of full cells. **a** Electrochemical behavior of Zn|GF|MnO_2_ cell. **b** CV curves of Zn|GF|MnO_2_ and Zn|UiO-66-GF-2.2|MnO_2_ cells. **c** EIS spectra and corresponding equivalent circuit diagram of Zn|GF|MnO_2_ and Zn|UiO-66-GF-2.2|MnO_2_ cells. **d** Rate performances of Zn|GF|MnO_2_ and Zn|UiO-66-GF-2.2|MnO_2_ cells. **e** Charge/discharge profiles of Zn|GF|MnO_2_ and Zn|UiO-66-GF-2.2|MnO_2_ cells at 0.3 A g^−1^. **f** GITT curves and **g** zinc-ions diffusion coefficients during discharging of Zn|GF|MnO_2_ and Zn|UiO-66-GF-2.2|MnO_2_ cells. **h** Cycling performances and CEs of Zn|GF|MnO_2_ and Zn|UiO-66-GF-2.2|MnO_2_ cells at 1.0 A g^−1^. **i** Cycling performances after resting for 24 h of three cells at 0.5 A g.^−1^

To evaluate the role of UiO-66-GF (Fig. 4a), full cells with α-MnO_2_ cathode (Fig. S10) were assembled. CV tests were performed to investigate the redox reaction and reversibility during the charge/discharge process. CV curves have the same shape and peak position, indicating that UiO-66 does not change the electrochemical process (Figs. 4b and S11). Two groups of redox peaks represent reversible (de)intercalation of hydrogen ions and zinc-ions from MnO_2_, respectively [58]. Compared with Zn|GF|MnO_2_ cell, Zn|UiO-66-GF-2.2|MnO_2_ cell has higher peak current density and smaller voltage gap, demonstrating a high electrochemical activity and a lower polarization [59]. Charge transfer resistance (*R*_ct_) of Zn|UiO-66-GF-2.2|MnO_2_ cell (133.6 Ω) is lower than those of Zn|GF|MnO_2_ (361.2 Ω) and Zn|UiO-66-GF-0.6|MnO_2_ cells (251.4 Ω) (Figs. 4c and S12), which confirms fast electrochemical kinetics [60]. Rate performance tests exhibit that the capacities of Zn|UiO-66-GF-0.6|MnO_2_ and Zn|UiO-66-GF-2.2|MnO_2_ cells basically return to the initial value after cycling, with better reaction kinetics than that of Zn|GF|MnO_2_ cell (Figs. 4d and S13) [61]. Overall, Zn|UiO-66-GF-2.2|MnO_2_ cell has higher capacity and more stable voltage platforms (Figs. 4e and S14). Furthermore, GITT measurements were performed to verify the effect of UiO-66 on zinc-ions transfer. Hysteresis voltage generated after intermittency of Zn|GF|MnO_2_ cell is almost twice that of Zn|UiO-66-GF-2.2|MnO_2_ cell, reflecting that electrochemical reaction resistance is smaller in Zn|UiO-66-GF-2.2|MnO_2_ cell (Fig. 4f) [62]. The zinc-ions diffusion coefficient (*D*_Zn_) of Zn|UiO-66-GF-2.2|MnO_2_ cell is higher than that of Zn|GF|MnO_2_ cell (1.30906 × 10^−10^ *vs*. 1.46465 × 10^−11^ cm^2^ s^−1^), which indicates UiO-66-GF-2.2 accelerates the transport of zinc ions at the interface of MnO_2_ (Fig. 4g) [63].

In addition, long-term cycling stabilities of cells at different current densities were also evaluated. Initial specific discharge capacity of Zn|UiO-66-GF-2.2|MnO_2_ cell is 198.5 mAh g^−1^ at 0.5 A g^−1^ along with 81.9% capacity retention after 1000 cycles, which is higher than those of Zn|UiO-66-GF-0.6|MnO_2_ cell (186.3 mAh g^−1^, 68.2%) and Zn|GF|MnO_2_ cell (165 mAh g^−1^, 58.5%) (Fig. S15). When current density increases to 1.0 A g^−1^, specific discharge capacity of Zn|GF|MnO_2_ cell decreases after only 200 cycles (Fig. 4h), while the Zn|UiO-66-GF-0.6|MnO_2_ is stable for 600 cycles (Fig. S16). Zn|UiO-66-GF-2.2|MnO_2_ cell still provides high discharge capacity after 1000 cycles (186.55 mAh g^−1^) along with a high capacity retention (85%). Meanwhile, zinc anode of Zn|UiO-66-GF-2.2|MnO_2_ cell does not exhibit significant surface changes after cycling and there are no obvious dendrites in cross-sectional SEM image (Fig. S17), reflecting UiO-66-GF which enables more uniform flux of zinc-ions, promoting uniform nucleation and deposition, and eliminating dendrites [64]. Zn|UiO-66-GF-2.2|MnO_2_ cell also demonstrates excellent self-discharge resistance, owing to the protection of the electrodes by UiO-66-GF-2.2 [65]. After resting for 24 h, Zn|UiO-66-GF-2.2|MnO_2_ cell maintains a sufficient discharge capacity due to self-discharge reduction [66]. Specific discharge capacity of Zn|UiO-66-GF-2.2|MnO_2_ (141 mAh g^−1^) is considerably higher than those of Zn|GF|MnO_2_ (31.5 mAh g^−1^) and Zn|UiO-66-GF-0.6|MnO_2_ (93.7 mAh g^−1^) cells after 400 cycles, implying that UiO-66-GF-2.2 can effectively improve the stability and service life of cells (Fig. 4i).

### Characterization of Zinc Anode during Repeated Cycling and Mechanism Analysis

**Fig. 5:**
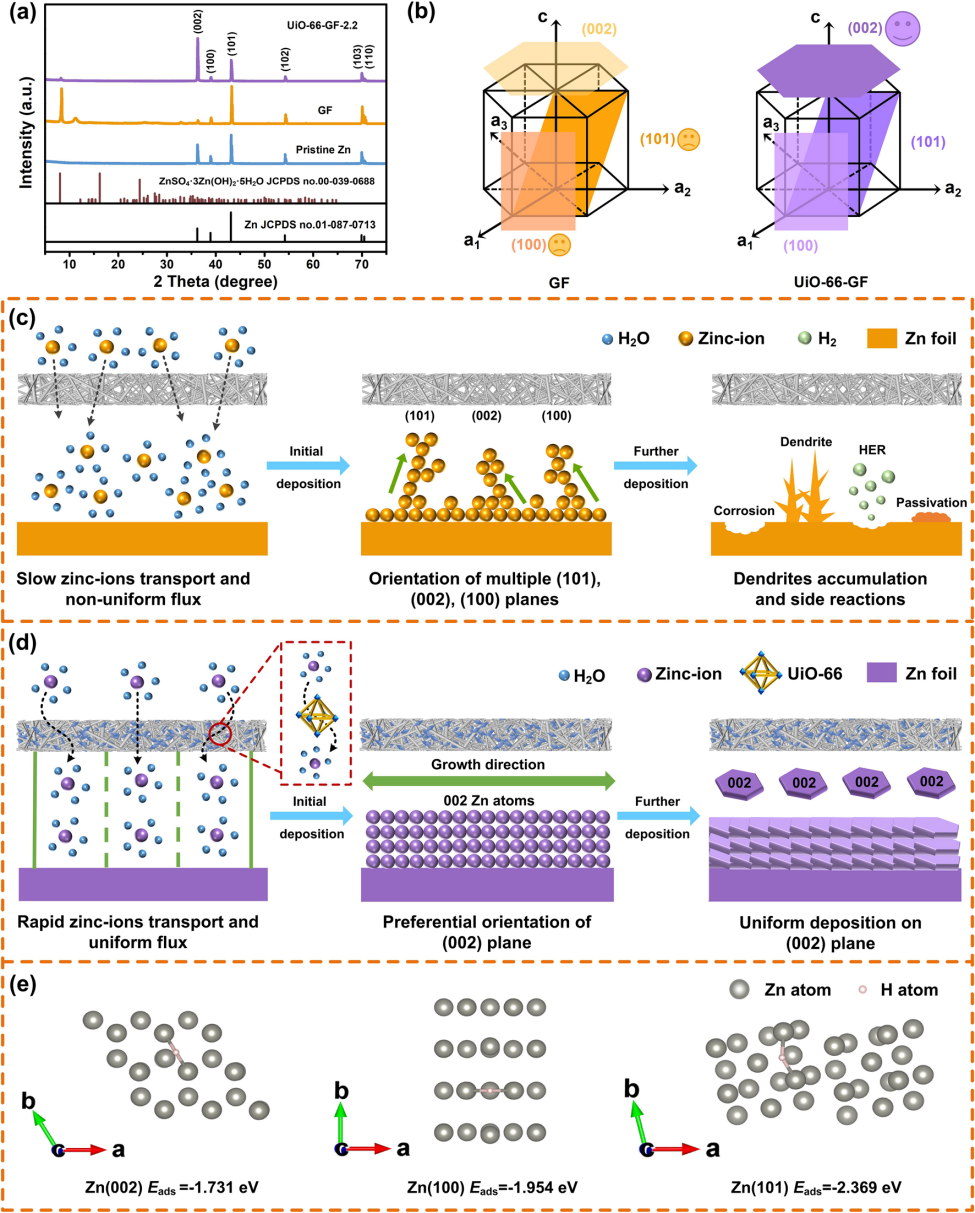
Characterization of zinc anode during repeated cycling and mechanism analysis. **a** XRD patterns of pristine Zn, Zn|GF|MnO_2_, and Zn|UiO-66-GF-2.2|MnO_2_ cells after cycling. **b** Schematic illustration of preferred orientations of Zn crystal plane. Mechanism comparison of the deposition processes for zinc anodes using **c** GF and **d** UiO-66-GF-2.2. **e** Adsorption energies between H and Zn (002), (100), and (101) crystal planes

To elucidate the mechanism of UiO-66-GF on the inhibition of zinc dendrites and corrosion resistance, XRD patterns of zinc anodes before and after cycling were measured (Fig. 5a). The diffraction intensity of (101) plane of zinc anode becomes higher in Zn|GF|MnO_2_ cell after cycling, indicating that zinc-ions tend to deposit in the vertical direction. However, zinc anode in Zn|UiO-66-GF-2.2|MnO_2_ cell shows a higher (002) preferred crystal orientation and a significantly higher (002)/(101) diffraction intensity ratio after cycling, proving that zinc-ions tend to deposit in the horizontal direction (Fig. 5b). The atomic arrangement and interfacial charge density distribution of the (002) and (101) planes is different. UiO-66-GF induces the growth of zinc-ions in (002) plane, culminating in dendrite-free zinc deposition (Fig. 5c-d) [67]. Further analysis of XRD data exhibits that UiO-66-GF-2.2 inhibits the formation of by-products such as ZnSO_4_·3Zn(OH)_2_·4H_2_O (JCPDS No. 00-009-0204), which also corresponds to EDX results (Fig. S18). In addition, adsorption energies between H and Zn (002), (100), and (101) crystal planes were analyzed using DFT calculations (Fig. 5e) [68]. Zn (002) plane demonstrates lower adsorption energy for H (− 1.731 eV) than that of (100) (− 1.954 eV) and (101) planes (− 2.369 eV), indicating a weaker adsorption of H by (002) plane, which is beneficial to improve corrosion resistance and suppress HER. The catalytic activities of HER on different crystal planes of zinc were evaluated by Δ*G*_H_. Theoretically, a large Δ*G*_H_ implies a high reaction overpotential of HER. Δ*G*_H_ of Zn (002) is 0.759 eV, which is larger than those of Zn (100) (0.536 eV) and Zn (101) planes (0.121 eV), indicating that the construction of Zn (002) plane helps inhibit the side reactions.

In general, HER not only leads to a local pH increase in the electrolyte, but also continuously consumes the water in the electrolyte, eventually leading to increases in the concentrations of OH^−^ and SO_4_^2−^. UiO-66-GF-2.2 demonstrates preferential orientation of (002) plane. DFT calculations exhibit a weaker adsorption of H by (002) plane. Therefore, UiO-66-GF-2.2 can effectively inhibit HER and further reducing the concentration of harmful anions in the electrolyte. Meanwhile, after using UiO-66-GF-2.2, the flux of zinc-ions becomes uniform, which makes the concentration of zinc-ions reach the surface of zinc anode more consistent. Uniform concentration of zinc-ions in the electrolyte near anode can reduce the generation of electrochemical corrosion products, thereby slowing down the generation of passivation layers, accelerating the rate of ion transfer, and enabling durable AZIBs.

## Conclusion

In conclusion, a separator (UiO-66-GF) modified by Zr-based MOF for robust AZIBs is successfully proposed. UiO-66 has large specific surface area and abundant pore structure, which enables the electrolyte to penetrate uniformly and effectively reduces the local current density. Benefiting from the well-filled interspace, the sufficient contact of zinc anode with electrolyte not only reduces the NOP, but also uniformizes the electric field distribution to tune the zinc deposition. UiO-66-GF effectively enhances transport ability of charge carriers and demonstrates preferential orientation of (002) crystal plane due to the uniform interfacial charge of (002) deposition, which is favorable for the growth of zinc along the horizontal direction. Furthermore, Zn|UiO-66-GF-2.2|Zn cell enables reversible plating/stripping with long cycle life over 1650 h at 2.0 mA cm^−2^, and excellent long-term stability with capacity retention of 85% is obtained for Zn|UiO-66-GF-2.2|MnO_2_ cell after 1000 cycles at 1.0 A g^−1^. This work provides a facile and economical approach for separator modifications, which is beneficial to further promote the practical application of AZIBs.

## Supplementary Information

Below is the link to the electronic supplementary material.

Supplementary file1 (PDF 1535 kb)
