# Supplementary material for: Metal–Organic Frameworks Functionalized Separators for Robust Aqueous Zinc-Ion Batteries
Source: Nanomicro Lett. 2022 Nov 9;14:218. doi: 10.1007/s40820-022-00960-z (PMC9646683; doi:10.1007/s40820-022-00960-z)
Supplement: Supplementary file 1 — Supplementary file1 (PDF 1535 kb) [file 40820_2022_960_MOESM1_ESM.pdf]

Supporting Information for

Metal-Organic Frameworks Functionalized Separators for Robust Aqueous Zinc-Ion Batteries

Yang Song[1, #], Pengchao Ruan[2, #], Caiwang Mao[1], Yuxin Chang[1], Ling Wang[1], Lei Dai[1], Peng Zhou[3], Bingan Lu[4], Jiang Zhou[2] *, and Zhangxing He[1, *]

[1] School of Chemical Engineering, North China University of Science and Technology, Tangshan 063009, P. R. China

[2] School of Materials Science and Engineering, Hunan Provincial Key Laboratory of Electronic Packaging and Advanced Functional Materials, Central South University, Changsha 410083, P. R. China

[3] Hunan Provincial Key Defense Laboratory of High Temperature Wear-Resisting Materials and Preparation Technology, Hunan University of Science and Technology, Xiangtan 411201, P. R. China

[4] School of Physics and Electronics, Hunan University, Changsha 410082, P. R. China

[#]Yang Song and Pengchao Ruan have contributed equally to this work.

*Corresponding authors. E-mail: zhou_jiang@csu.edu.cn (J. Zhou); zxhe@ncst.edu.cn (Z. He)

Supplementary Figures

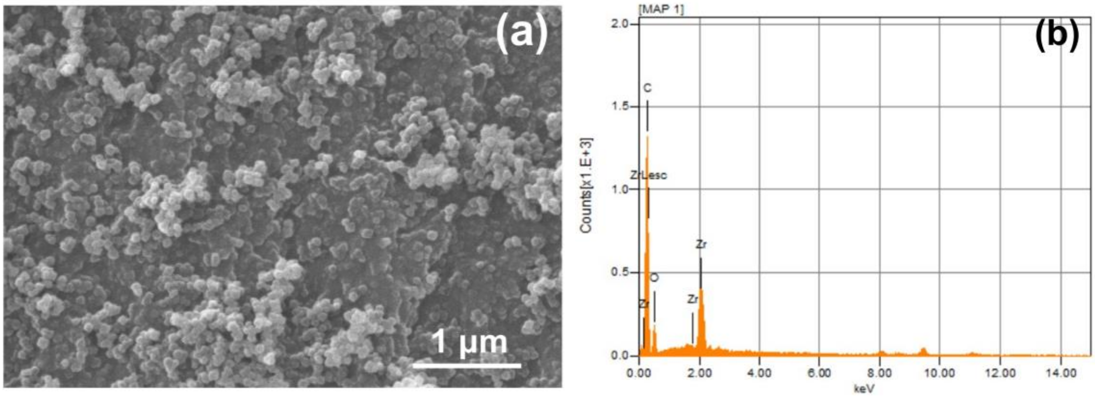

Fig. S1 (**a**) Surface SEM image of UiO-66. (**b**) EDX spectrum of UiO-66

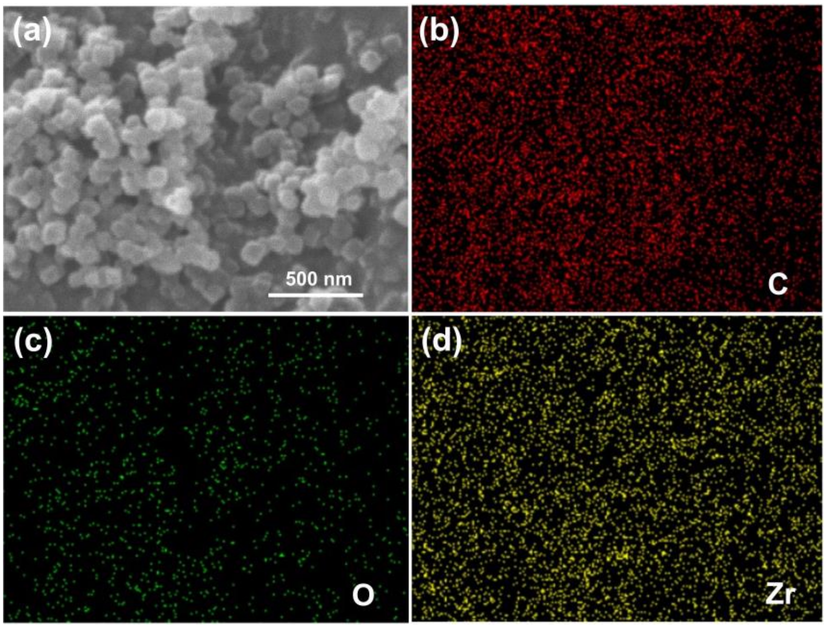

Fig. S2 (**a**) SEM and element mapping images of UiO-66 for (**b**) C, (**c**) O, and (**d**) Zr

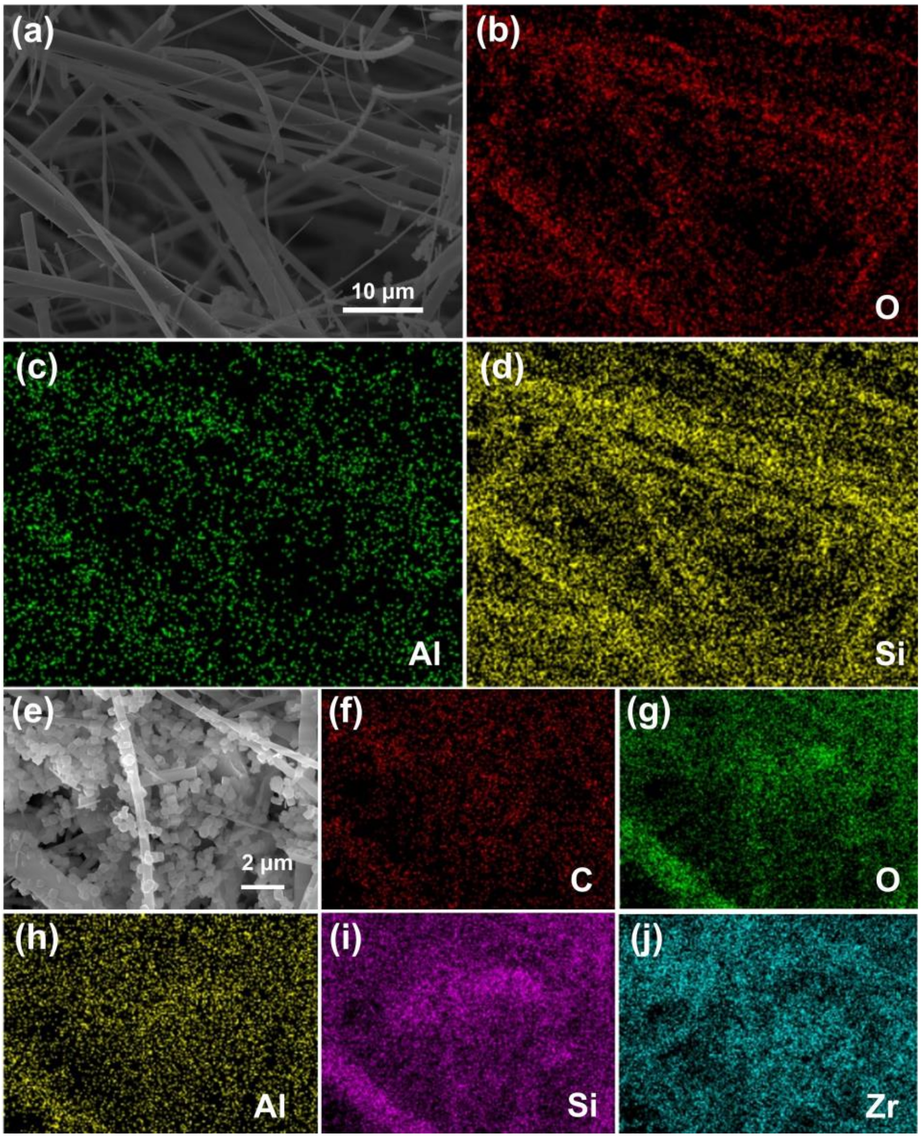

Fig. S3 (**a**) SEM and element mapping images of GF for (**b**) O, (**c**) Al, and (**d**) Si. (**e**) SEM and element mapping images of UiO-66-GF-0.6 for (**f**) C, (**g**) O, (**h**) Al, (**i**) Si and (**j**) Zr

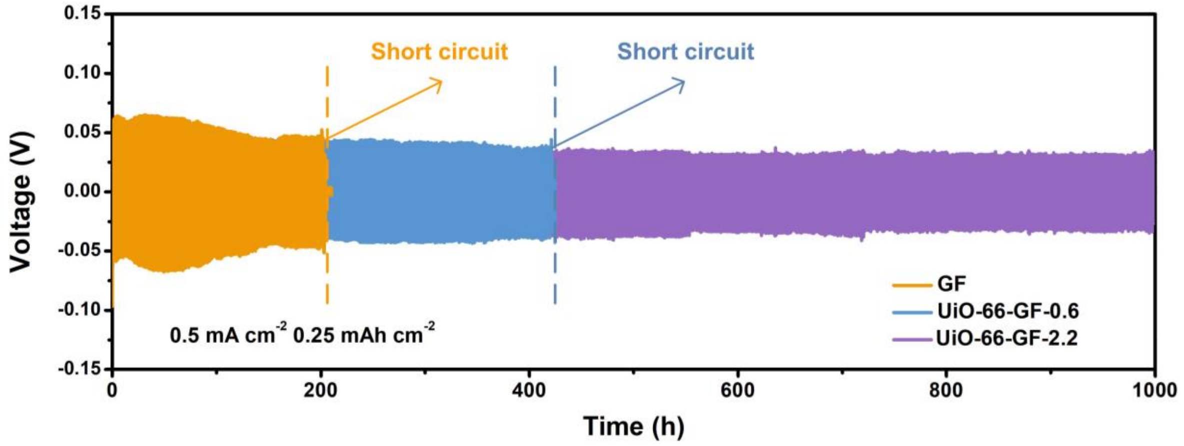

Fig. S4 Galvanostatic charge/discharge cycling voltage profiles of three cells at current densities of 0.5 mA cm^{-2} for 0.25 mAh cm^{-2}

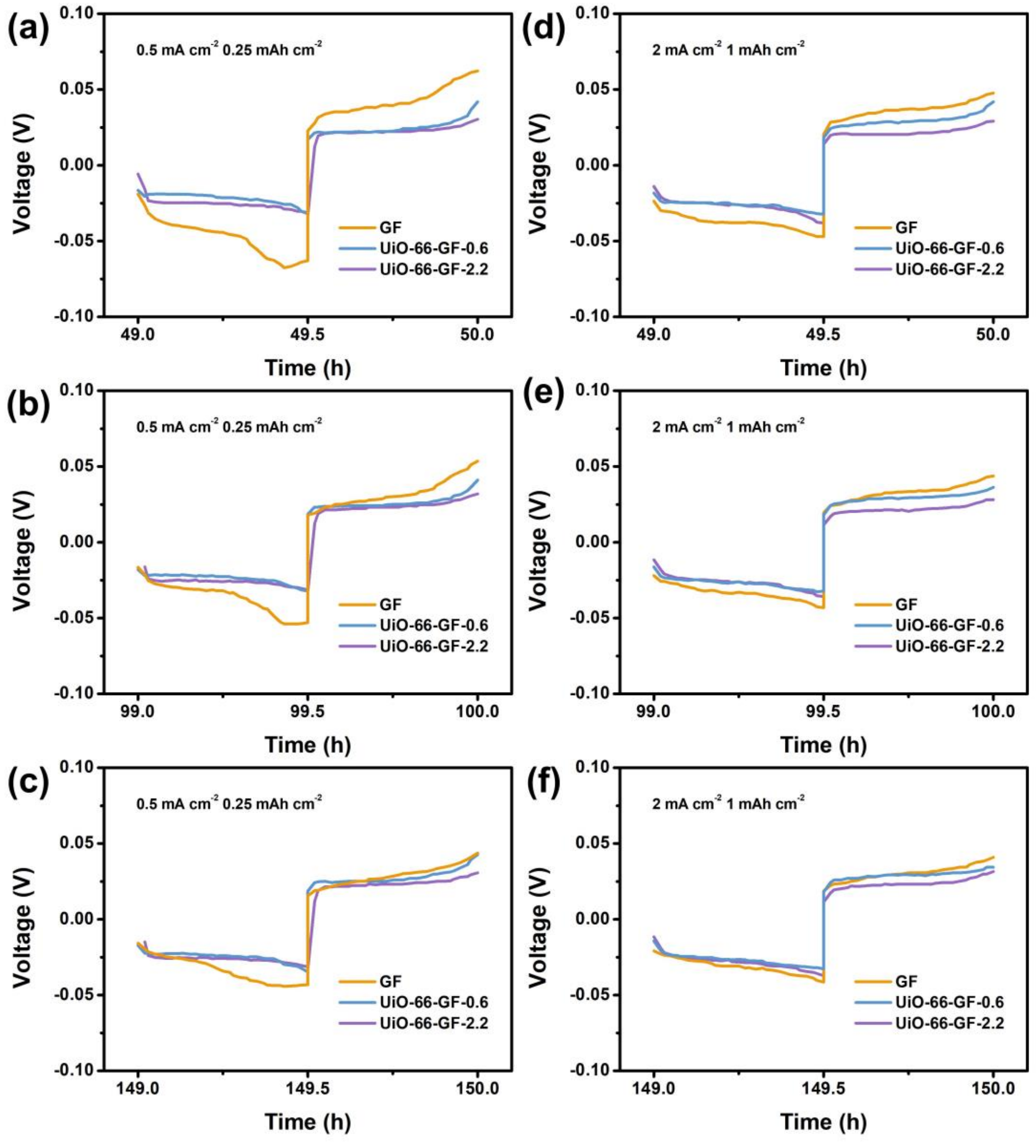

Fig. S5 Hysteresis voltage of three cells at current densities of 0.5 mA cm^{-2} for 0.25 mAh cm^{-2}, comparison of during various (**a**) 50th, (**b**) 100th, (**c**) 150th cycle periods. Hysteresis voltage of three cells at current densities of 2 mA cm^{-2} for 1 mAh cm^{-2}, comparison of during various (**d**) 50th, (**e**) 100th, (**f**) 150th cycle periods

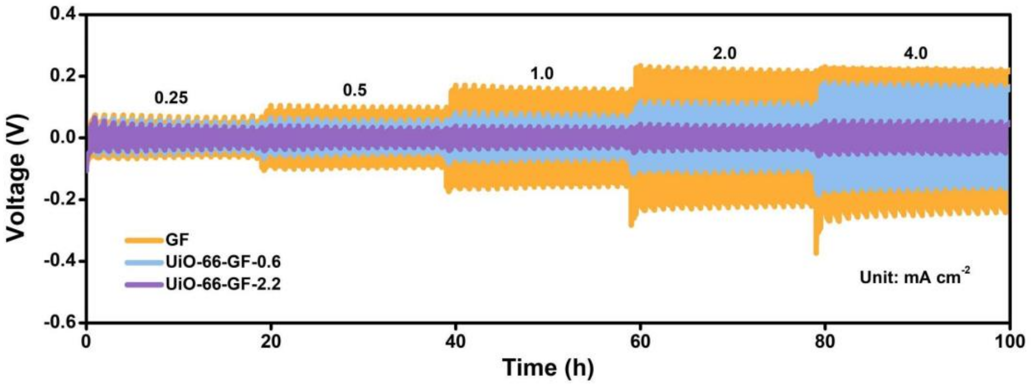

Fig. S6 Rate performances of Zn|GF|Zn, Zn|UiO-66-GF-0.6|Zn, and Zn|UiO-66-GF-2.2|Zn cells

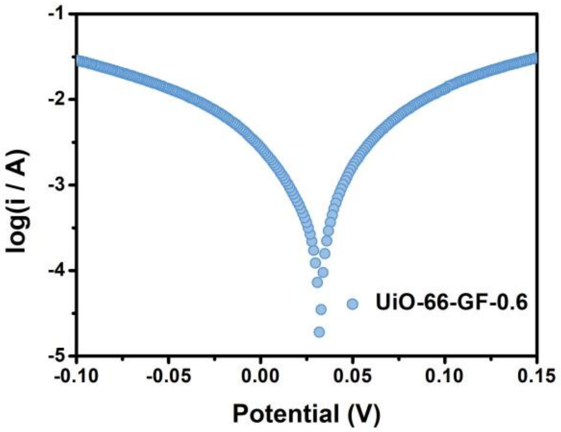

Fig. S7 Linear polarization curves presenting the corrosion on Zn|UiO-66-GF-0.6|Zn cell

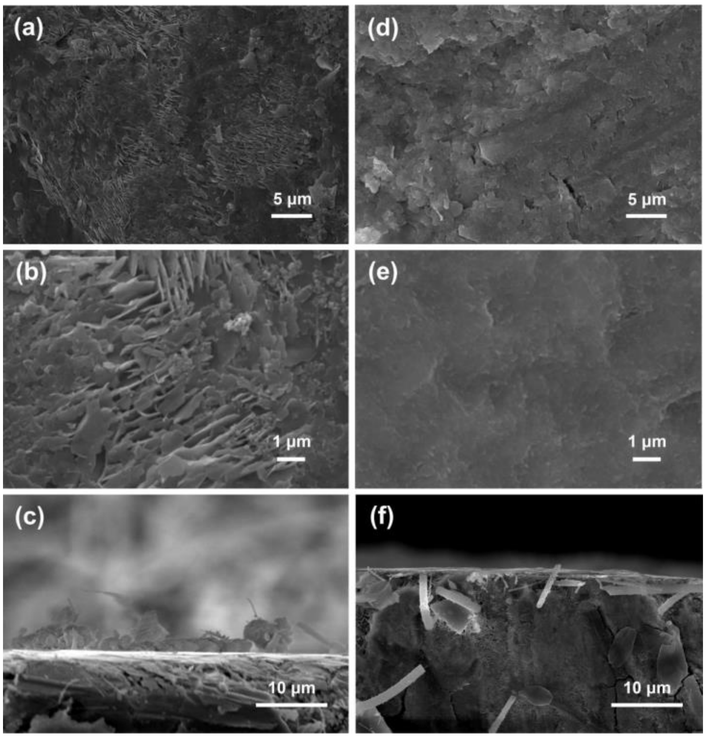

Fig. S8 (**a**) and (**b**) SEM images of Zn|GF|Zn cell after cycling on zinc anode surface at different magnifications. (**c**) Cross-sectional SEM image of Zn|GF|Zn cell after cycling on zinc anode. (**d**) and (**e**) SEM images of Zn|UiO-66-GF-2.2|Zn cell after cycling on zinc anode surface at different magnifications. (**f**) Cross-sectional SEM image of Zn|UiO-66-GF-2.2|Zn cell after cycling on zinc anode

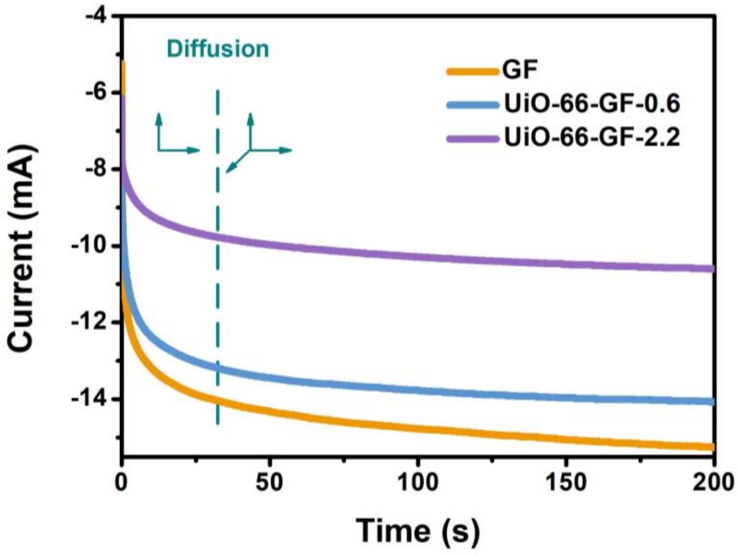

Fig. S9 CA of Zn|GF|Zn, Zn|UiO-66-GF-0.6|Zn, and Zn|UiO-66-GF-2.2|Zn cells

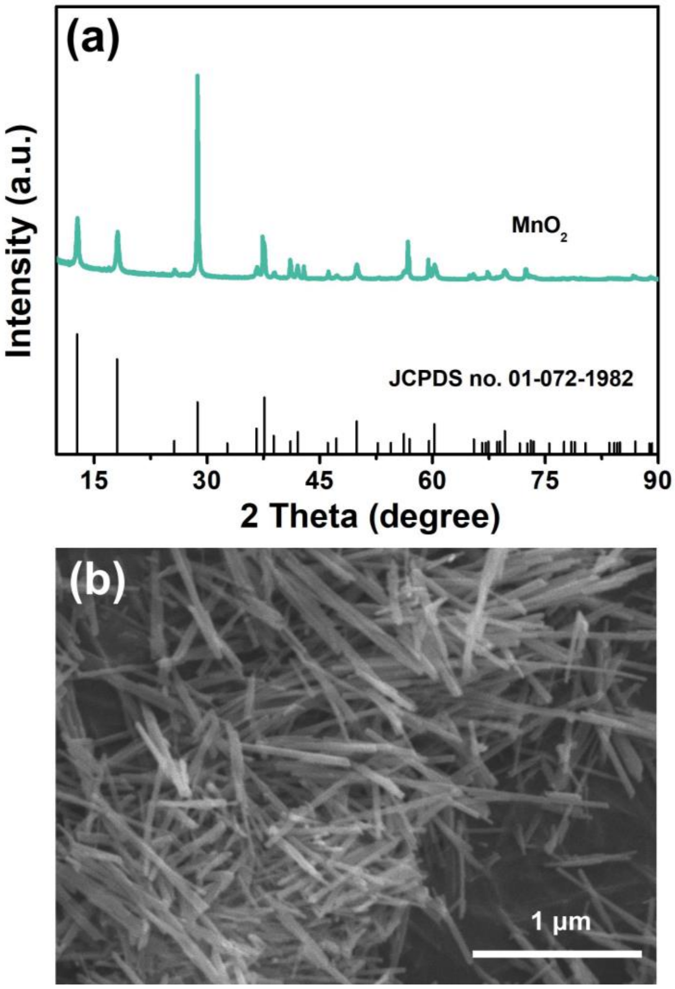

Fig. S10 (**a**) XRD pattern for MnO_2. (**b**) SEM image of MnO_2

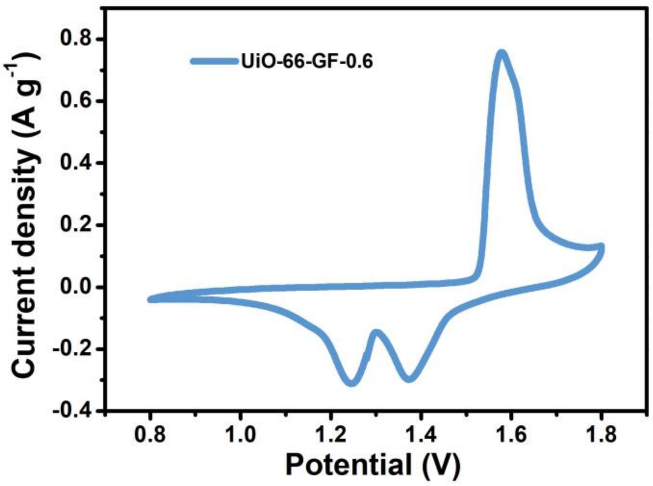

Fig. S11 CV curve of Zn|UiO-66-GF-0.6|MnO_2 cell

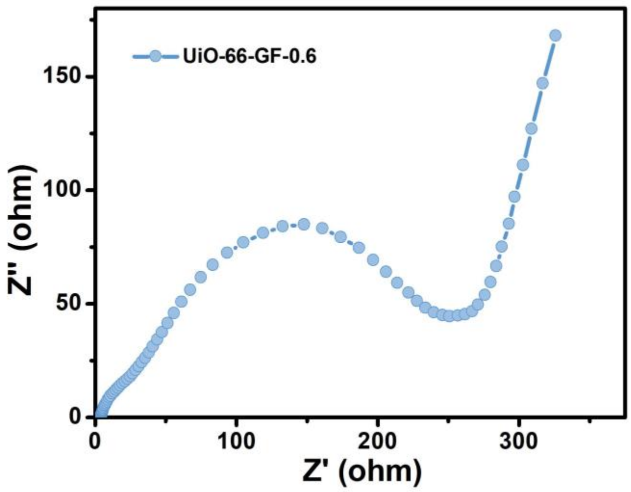

Fig. S12 EIS spectrum of Zn|UiO-66-GF-0.6|MnO_2 cell

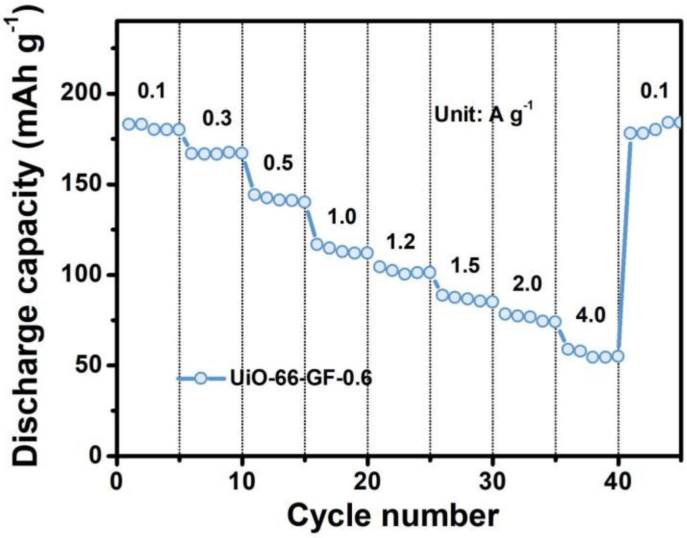

Fig. S13 Rate performance of Zn|UiO-66-GF-0.6|MnO_2 cell

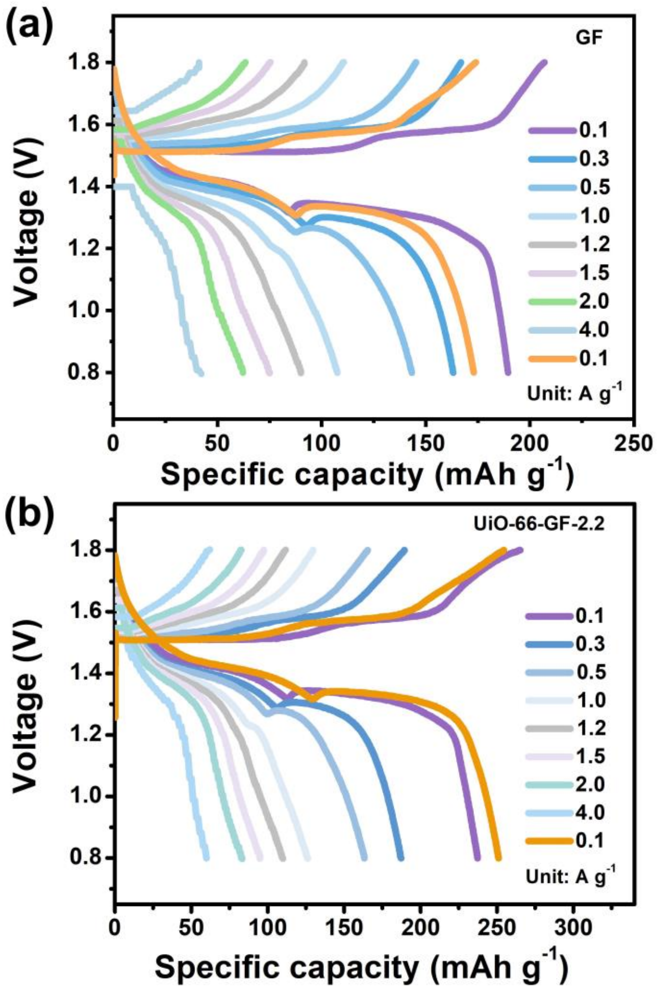

Fig. S14 Charge/discharge profiles of (**a**) Zn|GF|MnO_2 and (**b**) Zn|UiO-66-GF-2.2|MnO_2 cells ranging from 0.1 to 4 A g^{-1}

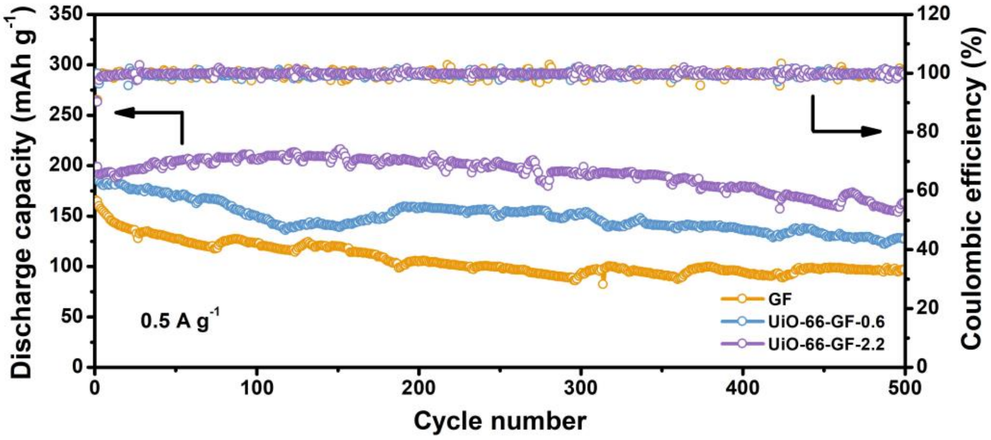

Fig. S15 Cycling performances and CEs of Zn|GF|MnO_2 and Zn|UiO-66-GF-2.2|MnO_2 cells at 0.5 A g^{-1}

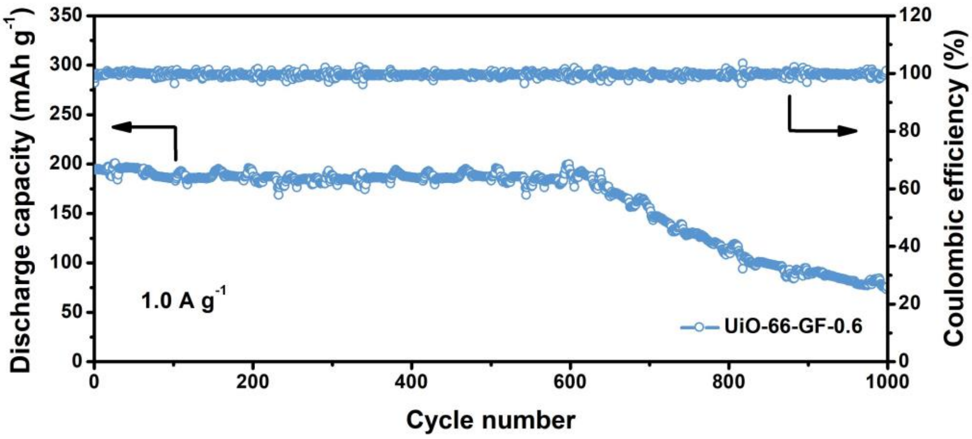

Fig. S16 Cycling performance and CE of Zn|UiO-66-GF-0.6|MnO_2 cell at 1.0 A g^{-1}

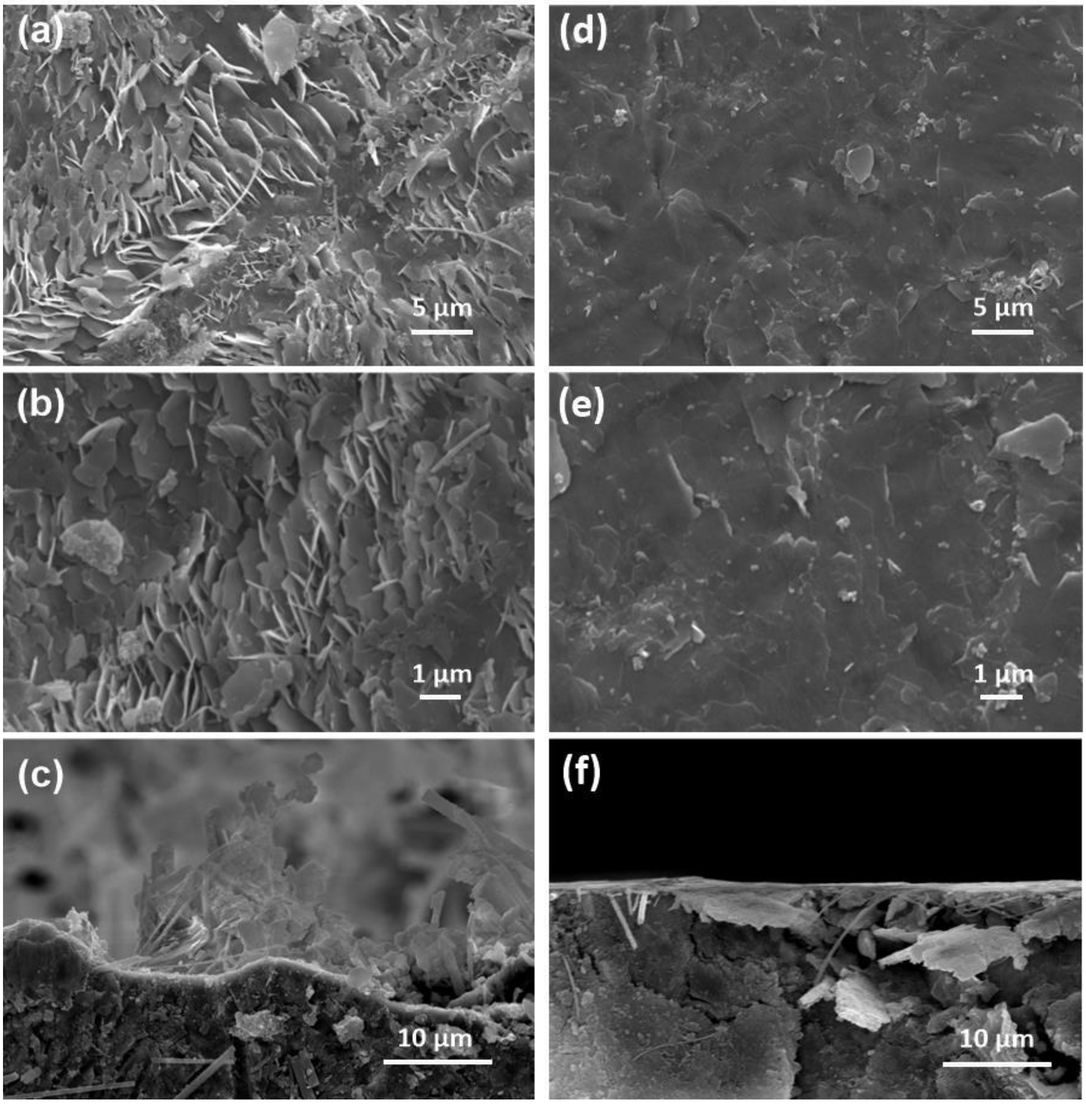

Fig. S17 (**a**) and (**b**) SEM images of Zn|GF|MnO_2 cell after cycling on zinc anode surface at different magnifications. (**c**) Cross-sectional SEM image of zinc anode in Zn|GF|MnO_2 cell. (**d**) and (**e**) SEM images of Zn|UiO-66-GF-2.2|MnO_2 cell after cycling on zinc anode surface at different magnifications. (**f**) Cross-sectional SEM images of zinc anode in Zn|UiO-66-GF-2.2|MnO_2 cell

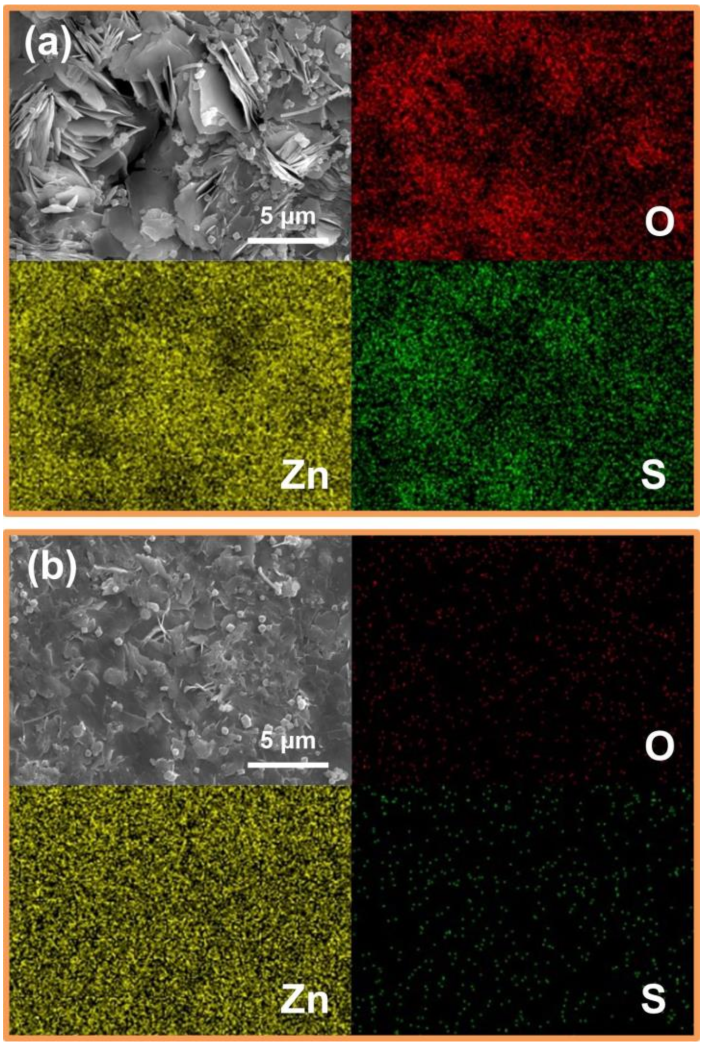

Fig. S18 SEM and element mapping images of (**a**) Zn|GF|MnO_2 and (**b**) Zn|UiO-66-GF-2.2|MnO_2 cells after cycling on zinc anode surfaces
